# Adolescent-to-Parent Violence: Psychological and Family Adjustment

**DOI:** 10.3389/fpsyg.2020.573728

**Published:** 2020-11-25

**Authors:** Dolores Seijo, María J. Vázquez, Raquel Gallego, Yurena Gancedo, Mercedes Novo

**Affiliations:** ^1^Psicología Organizacional, Jurídica Forense y Metodología de las Ciencias del Comportamiento, Facultad de Psicología, University of Santiago de Compostela, Santiago de Compostela, Spain; ^2^Facultad de Ciencias de la Educación y del Trabajo Social, University of Vigo, Ourense, Spain; ^3^Unidad de Psicología Forense, Facultad de Psicología, University of Santiago de Compostela, Santiago de Compostela, Spain

**Keywords:** adolescent-to-parent violence, parenting style, family system, maladjustment, victimization, childcare

## Abstract

Adolescent-to-Parent Violence (APV) or Child-to-Parent Violence (CPV) is a specific form of violence that has remained inconspicuous until recently, but is becoming a mounting social issue and is increasingly the focus of scientific research. Of the variables related to APV, the study assessed the characteristics of the family system and its relationship to the psychosocial adjustment of adolescents, an aspect scarcely examined in the literature. Thus, a field study was performed on a community sample of 210 adolescents aged 12–17 years (51.4% girls) who were assessed on measurements of APV, parenting (parental socialization), victimization, and psychological adjustment (personal, family, and school). The results revealed higher rates of psychological APV, and no gender effects in violence exercised against either parent. The adolescents involved in APV exhibited a greater psychological maladjustment in the different areas under analysis. Moreover, adolescents engaging in psychological APV reported a parental socialization style characterized by severe strictness and supervision in comparison to non-aggressors not implicated in psychological APV. Finally, adolescents exercising APV who were victimized by their parents showed more psychological, personal, and school maladjustment. These results have implications for needs analysis and the planning of community prevention strategies.

## Introduction

Antisocial behavior is a key issue in the field of Legal and Forensic Psychology (Arce et al., 2011). One of its expressions is adolescent-to-parent violence (APV, also known as child-to parent violence), a specific form of violence that has remained inconspicuous for decades (Ibabe, 2019), but was brought into the limelight in recent years owing to the rise in the number of cases and the severe impact on the entire family system (Holt, 2016; Del Hoyo-Bilbao et al., 2020). Owing to the social and legal involvements involved, the international scientific community is gradually shifting its focus toward this phenomenon but the number of specific APV studies still remains scarce (Gámez-Guadix and Calvete, 2012; Lyons et al., 2015). A recent systematic review (Simmons et al., 2018) has highlighted that the variations in the samples employed in previous studies and the plurality of definitions and measurements accounted for the discrepancies reported in the scientific literature (Gallego et al., 2019; Cortina and Martín, 2020; Loinaz and de Sousa, 2020).

According to the definition of APV, the data available on world prevalence rates revealed variations ranging from 5 to 21% for physical violence and higher rates of 33–93% for psychological violence (Simmons et al., 2018). In Spain, most field studies have estimated prevalence rates of 21% for physical violence (Ibabe et al., 2013; Calvete et al., 2014; Ibabe and Bentler, 2016), and rates of 33–93% for psychological and emotional violence (Calvete et al., 2013; Ibabe, 2015; Ibabe and Bentler, 2016; Cortina and Martín, 2020). This high variability suggests the existence of moderators underlying the relationship. Thus, prevalence rates depend on the sample employed, with boys vastly outnumbering girls in judicial samples (Armstrong et al., 2018) by around 59–87% (Simmons et al., 2018), whereas in normalized student or community samples, gender differences almost vanished (Loinaz et al., 2020). In relation to the type, violence, physical, or psychological, most of the studies on community and student samples found no significant gender differences in APV (Loinaz et al., 2020), whereas other studies reported girls exercised more psychological violence (Calvete et al., 2013; Rosado et al., 2017). In clinical and judicial samples, physical violence was mainly exercised by boys (Armstrong et al., 2018; Cortina and Martín, 2020), owing to the seriousness of the APV offense, this entailed a higher probability of custodial sentences, whereas girls were mainly involved in psychological violence. Nevertheless, other studies have found that girls in custody can also resort to severe forms of APV involving physical violence (Condry and Miles, 2014; Simmons et al., 2018).

Regarding the victims, several studies have shown mothers are more often the target of APV than fathers (Edenborough et al., 2008; Condry and Miles, 2014; Lyons et al., 2015) whilst other studies have found no significant differences between either parent (Loinaz et al., 2020), particularly in long-term violence (Calvete et al., 2013). Gender differences have also been related to types of violence, physical or psychological, with most physical violence being exercised by boys (Simmons et al., 2019), whereas mothers tend to be the target of psychological violence (Ibabe and Jaureguizar, 2010).

Family variables have gradually became the focus of research (Loinaz et al., 2018; Beckmann, 2020; Del Hoyo-Bilbao et al., 2020). Parenting styles have been linked to APV (Maccoby and Martin, 1983), in particular with authoritarian parenting styles in community and judicial samples, and a permissive parenting style in community, clinical, and offender samples (Simmons et al., 2018). Whereas parenting style is a key factor in the child’s evolutionary process, during adolescence, it is crucial as it decisively influences attitudes and behavior (Cutrín et al., 2018). Research on the impact of different socialization styles has identified several factors linked to an adolescent’s adaptation. Whereas a democratic style predicted greater psychosocial development, self-esteem, and academic achievement (Ibabe, 2015), an authoritarian, permissive, or neglectful style had negative outcomes for adolescents such as somatic symptoms, emotional stress, and antisocial and/or deviant behavior (Lamborn et al., 1991; Contreras and Cano, 2014; Ibabe, 2015; Suárez-Relinque et al., 2019). The influence of parenting styles on antisocial behavior have identified poor supervision and discipline as a crucial risk factor for this type of behavior in adolescence (Perez-Gramaje et al., 2020). The parents of APV adolescents were reluctant to impose discipline when children misbehaved, and showed lower levels of affect and support (Gámez-Guadix et al., 2012; Ibabe et al., 2013; Calvete et al., 2015). Recent studies support the relevance of affection and attachment in family relations (Beckmann et al., 2017; Curtis et al., 2019; Suárez-Relinque et al., 2019) with girls and boys who failed to receive affection adopting inappropriate problem-solving strategies, including APV (Gámez-Guadix et al., 2012; Cortina and Martín, 2020), whilst boys and girls exposed to coercive parental behavior appear to be at an increased risk of developing behavioral problems (Pasalich et al., 2011). Complementarily, several empirical studies have shown that affective warmth, emotional nurturance, and support giving were protective factors against the risk of violent behavior in children and adolescents (Jiménez-García et al., 2019; Suárez-Relinque et al., 2019; Cortina and Martín, 2020).

Furthermore, numerous studies on direct and vicarious victimization in childhood as a precipitator of APV have underscored the hypothesis of bidirectionality, i.e., parents-to-child violence predict child-to-parent violence (Routt and Anderson, 2011; Contreras and Cano, 2016; Del Hoyo-Bilbao et al., 2020). In a recent meta-analysis, Gallego et al. (2019) concluded that the probability of developing APV among adolescents victimized by their parents was 71% higher than in non-victimized adolescents under different conditions (community or judicial population, type of violence: physical or psychological, and type of victimization: direct or vicarious). Though bidirectionality has been well established, the same cannot be said of the adjustment in each of the significant areas of adolescents engaged in APV who were victimized by their parents (Haw, 2010; Novo et al., 2019; Contreras et al., 2020).

Thus, the aims of this study on the family system of adolescents engaged in APV were threefold: (1) to evaluate the personal and school psychological adjustment of adolescents involved in APV; (2) to assess parenting (parental socialization styles) as informed by the self-reports of APV and non-APV adolescents; and (3) to compare psychosocial adjustment in victimized adolescents and victimized adolescents who also engaged in APV.

## Materials and Methods

### Participants

A total of 210 adolescents, age range 12–17 years (*M* = 13.21, *SD* = 0.94), from secondary schools in Galicia (Spain), participated in this study. The sample was balanced in terms of gender (107 girls, 51.4%) χ^2^(1) = 0.08, *ns*. As for the family structure informed by the participants, 79% were intact families and 17.7% were modifications to the original family unit, the main reasons were parental separation or divorce (14.8%), work (1.9%), or death (0.9%). In relation to schooling, 13% were first, 20% were second, and 67% were third-year Compulsory Secondary Education students.

### Measurement Instruments

For measuring APV, the Conflict Tactics Scale: Parent-Child Version (CTS-PC) (Straus and Fauchier, 2007) was administered. The instrument consists of six items, three for measuring physical violence (e.g., I slapped or punched my father/mother) and three for psychological violence (e.g., I shouted at my mother/father), answered on a three-point Likert response scale from *never* (0) to *often* (2) referring to the last year. The CTS-PC is an adapted version of the CTSCP scale but the directionality of the behavior has been modified. The response format is in line with the original scale, taking as a reference period the previous year. The reliability (Cronbach’s alpha) of the scale was 0.63 for mothers and 0.59 for fathers.

The adjustment of the adolescents was evaluated using the Spanish adaptation of the Children and Adolescents Behavior Evaluation System (González et al., 2004) S3 self-report. This instrument evaluates several aspects of behavior and personality, including both positive (adaptive) and negative (clinical) dimensions. The questionnaire comprises 14 scales grouped into clinical and adaptive scales. Moreover, it includes an F Index (measuring the negative tendency to respond negatively to adolescent behavior) an L Index (tendency for the adolescent to respond too positively), a Response Consistency Index, and a Response Pattern Index. As for the reliability of the scales, internal consistency was estimated to range from 0.70 to 0.90.

The Parental Socialization Scale in Adolescence ESPA-29 (Escala de Socialización Parental en la Adolescencia ESPA-29; Musitu and García, 2001) was employed to assess parental socialization styles. This scale evaluates parental socialization styles in different representative scenarios. Children evaluate their father and mother separately in 29 situations. As for the procedure, 13 of the 29 situations are evaluated with the affect and indifference subscales. The remaining 16 situations are evaluated by the dialogue subscale (“speak to me”), neglect subscale (“s/he doesn’t care”), psychological strictness subscale (“s/he tells me off”), physical strictness subscale (“s/he hits me”), and privation subscale (“I’m not allowed something”). Each scale has a 4-point scoring format (1, *never*; 2, *sometimes*; 3, *often*; and 4, *always*). The score for the Acceptance/Involvement dimension is obtained from the dialogue, affect, and neglect subscales, whereas the score of the Strictness/Supervision dimension was calculated as the mean of the mean scores of the strictness, psychological strictness, and privation subscales. Internal consistency (Cronbach’s alpha) was of 0.97 for the Acceptance/Involvement dimension, and of 0.96 for Strictness/Supervision.

For measuring victimization, the Parent-Child Conflict Tactics Scales (CTSPC) (Straus et al., 1998) was administered. This scale consists of six items measuring the frequency to which children suffer physical and/or psychological abuse from their parents, with a three-point response format: 0 (*never*), 1 (*sometimes*), and 2 (*often*). Each item is responded twice, one referring to the mother and the other, the father. In the present study, the internal consistency of the scale was an α of 0.83 for the father and an α of 0.78 for the mother.

### Procedure and Design

A filed study with a community sample was designed to quantify the prevalence of APV, the deviation from normativity of child-to parent offenders in personal and school psychological adjustment; the mean comparison between offenders and non-offenders in parental socialization styles; the association between parent-to child violence and child-to parent violence; and the effects of parent-to child victimization in the psychological, personal, and school and adjustment.

A community sample was gathered by accidental sampling from public schools in Galicia (northwest of Spain). In all schools, informed consent was obtained from the parents and tutors of the adolescents prior to inclusion in the study.

Participants were administered the questionnaires in two sessions in small groups in their usual classrooms. Participants were assured their data would remain anonymous and confidential in accordance with the Spanish data protection law (Ley Orgánica 3/2018 de Protección de Datos Personales y Garantía de los Derechos Digitales).

### Data Analysis

Contingency tables were used to summarize the categorical variables, and the chi-square test was ran to analyze statistical differences. For the continuous variables, the comparison of the means between groups was performed using the Student’s *t*-test for independent samples. The magnitude of the effect sizes was interpreted in terms of the Probability of Superiority of the Effect Size (PSES; Monteiro et al., 2018), a quantitative estimate of the effect-size i.e., probability of the superiority of the observed effect size in relation to all possible.

The identification of cases of adolescents who had exercised APV was in accordance with the “Zero tolerance” criterion enshrined in law and the directives of internationally recognized institutions and bodies such as the European Parliament Resolution on Zero Tolerance (Recommendation A4-0250/97, Resolution 2017/2897). In order to apply this criterion, participants were classified according to the CTS-PC Scale responses into individuals who had committed APV (raw score ≥ 1) vs. those who had not (raw score = 0).

## Results

### Frequency

The analysis of the frequency of APV according to typology (i.e., psychological or physical), and the parent’s gender revealed that psychological violence was employed both toward the mother (108 adolescents, 51.4%) and the father (109 adolescents, 51.9%), whereas the frequency of physical violence was 1.9% (4) for both parents. The results showed no gender differences between girls and boys in physical and psychological violence toward either parent, father, and mother alike (see Tables 1, 2).

**TABLE 1:** Independent-samples *t*-test on type of violence against parent for the factor aggressor’s gender (boy vs. girl).

Variable	*t*	*p*	*M*_*M*_	*SD*_*M*_	*M*_*F*_	*SD*_*F*_	*d*(PS_*ES*_)
APV physical father	0.44	0.792	0.48	1.01	0.39	1.14	0.08 (0.040)
APV psychological father	0.35	0.806	1.32	1.37	1.25	1.48	0.00 (0.000)
APV total father	0.57	0.942	1.68	1.94	1.51	2.21	0.08 (0.040)

**df(128); M_*M*_, mean of the boys’ group in APV; SD_*M*_, standard deviation of the boys’ group; M_*F*_, mean of the girls’ group in APV; SD_*F*_, standard deviation of the girls’ group; d, Cohen’s; PS_*ES*_, Probability of Superiority of the Effect Size.**

**TABLE 2:** Independent-samples *t*-test on type of violence against the mother for the factor aggressor’s gender (boy vs. girl).

Variable	*t*	*p*	*M*_*M*_	*SD*_*M*_	*M*_*F*_	*SD*_*F*_	*d*(PS_*ES*_)
APV physical mother	−0.53	0.416	0.08	0.55	0.01	0.10	0.18 (0.103)
APV psychological mother	−0.58	0.085	1.23	1.36	1.36	1.64	0.09 (0.048)
APV total mother	−0.67	0.168	1.38	1.95	1.58	2.19	0.10 (0.056)

**df(128); M_*M*_, mean of the boys’ group in APV; SD_*M*_, standard deviation of the boys’ group; M_*F*_, mean of the girls’ group in APV; SD_*F*_, standard deviation of the girls’ group; d, Cohen’s; PS_*ES*_, Probability of Superiority of the Effect Size.**

### Personal and School Psychological Adjustment

Thereafter, the psychological, personal, and school adjustment of adolescents engaged in growing violence in different significant spheres and/or areas was analyzed. Thus, the variable (APV vs. no APV) was recoded according to the “Zero tolerance” criterion (see section “Data Analysis”), with a total of 109 adolescents (51.9%) self-reporting growing psychological violence toward the father and 108 (51.4%) toward the mother. In relation to physical APV, four adolescents (1.9%) informed of violence toward the father and/or mother.

After recoding the psychological violence variable, the scores obtained by participants on the BASC scales were contrasted with the test value, i.e., the mean of the normative sample being obtained from the scoring manual of the instrument (González et al., 2004; Table 3).

**TABLE 3:** One sample *t*-tests of psychological APV toward mother on BASC dimensions.

Variables	*t*	*M*_*APV*_	*SD*_*APV*_	*M*_*tv*_	*d*(PS_*ES*_)
**Psychological adjustment**					
Atypicality	2.59**	5.45	7.01	3.7	0.32 (0.182)
Locus of control	1.58	4.06	3.04	3.6	0.16 (0.088)
Somatization	3.55***	1.71	2.09	1.0	0.41 (0.228)
Social stress	2.09*	3.36	3.27	2.7	0.22 (0.128)
Anxiety	3.07**	8.37	2.27	7.4	0.34 (0.190)
Depression	3.52***	2.91	3.25	1.8	0.39 (0.220)
Sense of inadequacy	4.05***	4.60	3.33	3.3	0.43 (0.332)
**Personal adjustment**					
Interpersonal relations	−2.15*	13.99	2.92	14.6	−0.24 (0.136)
Relations with parents	−0.99	7.58	2.19	7.8	−0.11 (0.088)
Self-esteem	−2.87**	5.89	2.51	6.6	−0.31 (0.174)
Self-confident	0.834	8.52	2.36	6.8	0.90 (0.632)
**School adjustment**					
Negative school Attitude	4.23***	3.11	2.94	1.9	0.45 (0.252)
Negative attitude teachers	4.46***	3.76	2.68	2.6	0.47 (0.362)
Sensation seeking	1.77	5.25	3.11	4.7	0.18 (0.104)
**GLOBAL INDEXES**					
Clinical maladjustment	2.67**	211.26	43.37	200	0.30 (0.583)
Personal adjustment	−2.76**	189.23	41.01	200.3	−0.31 (0.587)
School maladjustment	3.49***	154.31	31.01	143.8	0.38 (0.606)
Emotional symptoms	2.69**	316.50	64.83	0.30	0.31 (0.583)

df(107); M_*APV*_, mean of the APV group; SD_*APV*_, standard deviation of the APV group; M_*tv*_, test value (mean of the normative sample); d, Cohen’s d; PS_*ES*_, Probability of Superiority of the Effect Size. ***p < 0.001, **p < 0.01, *p < 0.05.

As shown in Table 3, in relation to psychological adjustment, adolescents engaged in psychological APV toward the mother scored significantly higher than the normative population in *Atypicality, Somatization, Social stress, Anxiety, Depression*, and *Sense of inadequacy*, as well as on the global *Clinical maladjustment* index, with greater maladjustment in all of the scales assessed. As for personal adjustment, significant differences were found in the *Interpersonal relations, and Self-esteem* scales and their global indexes, with low adjustment values for adolescents exercising growing violence. As for the school area, the results were significant in the *Negative attitude toward teachers, Negative attitude toward school* scales, and global *School Maladjustment* index, revealing APV adolescents exhibited more hostile resentment or dissatisfaction toward school and teachers as compared to the normative population. The probability of superiority of the effect sizes for the statistical significance results (see Table 3) ranged from 36.2% (Negative attitudes toward teachers) to 12.8% (Social stress) i.e., the magnitude of the effect size is greater than 34.8–12.8% of all possibilities.

Adolescents involved in psychological APV toward the father showed a significantly higher psychological maladjustment as compared to the normative population on the *Atypicality, Somatization, Social stress, Anxiety, Depression, Sense of inadequacy* scales, and global index of *Clinical maladjustment.* As for the area of Personal adjustment, *Self-esteem* and *Interpersonal relations*, and the global index of *Personal adjustment* revealed significant differences, with adolescents engaged in growing violence showing a lower self-esteem and higher personal maladjustment. In terms of school, APV adolescents scored significantly higher on the *Negative attitude toward teachers, Negative attitude toward school* scales, and *School maladjustment* index, with more hostile thoughts and a generalized rebuff toward the school, teachers, and structure of education. The magnitude of the statistically significant effect sizes (see Table 4) ranged from 34.8% (Negative attitudes toward teachers) to 12.8% (Social stress) i.e., the magnitude of the effect size is greater than 34.8–12.8% of all possibilities.

**TABLE 4:** One sample *t*-tests of psychological APV adolescents toward father on BASC dimensions.

Variables	*t*	*M*_*APV*_	*SD*_*APV*_	*M*_*tv*_	*d*(PS_*ES*_)
**Psychological adjustment**					
Atypicality	2.72**	5.50	6.91	3.7	0.33 (0.182)
Locus of control	1.60	4.05	2.95	3.6	0.00 (0.000)
Somatization	3.32***	1.62	1.96	1.0	0.37 (0.206)
Social stress	2.02*	3.34	3.30	2.7	0.22 (0.128)
Anxiety	2.88**	8.28	3.19	7.4	0.27 (0.150)
Depression	3.56***	2.95	3.36	1.8	0.39 (0.304)
Sense of inadequacy	4.67***	4.78	3.30	3.3	0.49 (0.376)
**Personal adjustment**					
Interpersonal relations	−2.75**	13.81	2.96	14.6	−0.30 (0.236)
Relations with parents	−1.28	7.52	2.20	7.8	−0.15 (0.080)
Self-esteem	−2.93**	5.87	2.54	6.6	−0.32 (0.182)
Self-confidence	0.767	8.37	2.28	6.8	0.85 (0.452)
**School adjustment**					
Negative attitude school	4.51***	3.10	2.76	1.9	0.46 (0.258)
Negative attitude teachers	4.70***	3.75	2.56	2.6	0.45 (0.348)
Sensation seeking	1.32	5.10	3.15	4.7	0.13 (0.072)
**Global indexes**					
Clinical maladjustment	2.58*	210.32	41.58	200.0	0.29 (0.228)
Personal adjustment	3.16*	187.33	42.55	200.3	−0.36 (0.282)
School maladjustment	3.50***	153.80	29.70	143.8	0.38 (0.296)
Emotional symptoms	2.88**	317.7	65.83	299.5	0.32 (0.296)

df(108); M_*APV*_, mean of the APV group; SD_*APV*_, standard deviation of the APV group; M_*tv*_, test value (mean of the normative sample); d, Cohen’s d; PS_*ES*_, Probability of Superiority of the Effect Size. ***p < 0.001, **p < 0.01, *p < 0.05.

### Parental Socialization Styles

The results showed that adolescents engaging in psychological APV toward the mother (see Table 5) informed of a parental socialization style characterized by little *Affect* and much *Indifference*, in comparison to a non-aggressor not engaging in psychological APV. The global dimensions of *Acceptance/Involvement and Strictness/Supervision* were significant, with *Acceptance/Involvement* being lower in APV adolescents as compared to non-aggressors; and higher on the *Strictness/Supervision* scale. The effect sizes of each of the global dimensions were small. In relation to the father, only the *Strictness/Supervision* dimension was statistically significant, with higher scores in adolescents involved in growing violence, than in non-aggressors. Nevertheless, the magnitude of the probability of superiority of the effect was small: 19.6% for affect, 19.8% for indifference; 19.0% for acceptance/involvement, and 17.4% for strictness/supervision.

**TABLE 5:** Independent-samples *t*-test on parenting (parental socialization styles) for the factor psychological APV.

Variables	*t*	*M*_*APV*_	*SD*_*APV*_	*M*_*n*–*APV*_	*SD*_*n*–*APV*_	*d*(PS_*ES*_)
**Mother (*n* = 173)**						
Dialogue	0.41	2.98	0.76	3.03	0.84	−0.06 (0.032)
Affect	2.28*	3.06	0.72	3.30	0.66	−0.35 (0.196)
Neglect	1.85	1.26	0.40	1.15	0.33	0.30 (0.166)
Indifference	2.36*	1.64	0.75	1.39	0.62	0.36 (0.198)
Physical coercion	−1.75	1.13	0.48	1.03	0.30	0.25 (0.142)
Privation	−1.03	1.97	0.66	1.86	0.75	0.16 (0.088)
Psychological coercion	−1.90	2.71	0.65	2.50	0.75	0.30 (0.166)
Acceptance/Involvement	2.23*	3.26	0.47	3.42	0.46	−0.34 (0.190)
Strictness/Supervision	−1.98*	1.94	0.43	1.80	0.49	0.31 (0.174)
**Father (*n* = 167)**						
Dialogue	0.57	2.81	0.79	2.87	0.78	−0.08 (0.048)
Affect	0.89	2.99	0.71	3.08	0.69	−0.13 (0.072)
Neglect	−1.58	1.29	0.40	1.20	0.34	0.24 (0.136)
Indifference	−1.28	1.57	0.56	1.46	0.60	0.19 (0.104)
Physical coercion	−1.80	1.13	0.33	1.04	0.32	0.28 (0.158)
Privation	−1.36	1.91	0.623	1.78	0.66	0.20 (0.112)
Psychological coercion	−1.22	2.55	0.66	2.43	0.61	0.19 (0.104)
Acceptance/Involvement	1.22	3.23	0.46	3.31	0.44	−0.18 (0.104)
Strictness/Supervision	−2.21*	1.88	0.43	1.74	0.40	0.34 (0.190)

df(171/165); M_*APV*_, mean of APV adolescent group; SD_*APV*_, standard deviation of APV adolescent group; M_*non–APV*_, mean of non-aggressor adolescent group; SD_*n*–*APV*_, standard deviation of non-aggressor adolescent group; d, Cohen’s d; PS_*ES*_, Probability of Superiority of the Effect Size. *p < 0.05.

### Experience of Child Victimization

APV and victimization variables were recorded in line with the previously mentioned “Zero tolerance” criterion to create only one variable, “adolescents engaged in APV,” with values 0 (absence of APV) and 1 (presence of APV). The results of this classification showed that 121 participants (57.6%) reported an instance of violent behavior toward parents in the last year vs. 73 (34.7%) who had not and 16 (7.6%), non-respondents. The same procedure was employed to quantify parent-to-child violence of participants to obtain only one variable of “victimization,” with only two values 0 (absence of victimization) vs. 1 (presence of victimization). A total of 174 adolescents (82.8%) reported an instance victimization, vs. 23 (10.9%) adolescents reporting no victimization, and 13 (6.1%) non-respondents. The results showed victimization was significantly associated to child-to-parent violence, χ^2^(1, *N* = 174) = 34.78, *p* < 0.001, that is, a relationship between being victimized by parents and being violent toward them, with a large effect size of φ = 0.426 (*PS*_*ES*_ = 0.541, i.e., the effect is greater than 54.1% of all possible effects).

The results (see Table 6) revealed that victimized APV adolescents showed a higher *maladjustment* in the global indexes (*Clinical maladjustment*, higher *School maladjustment*, and lower *Personal adjustment*) with moderate magnitude effects sizes. Moreover, most of the scales in each of the areas under analysis were significant (except *Anxiety, Interpersonal relations, and Negative attitude toward school and teachers*), which indicated a greater *maladjustment* in victimized APV adolescents with a probability of superiority of the effect size from 39.8% (Personal adjustment) to 22.8% (Somatization).

**TABLE 6:** Independent-samples *t*-test on the BASC dimensions for the factor victimization.

Variables	*t*	*M*_*APV*_	*SD*_*APV*_	*M*_*n*–*APV*_	*SD*_*n*–*APV*_	*d*(PS_*ES*_)
**Adjustment psychological**						
Atypicality	−2.53*	4.87	3.53	3.08	3.18	0.51 (0.282)
Locus of control	−2.13*	3.98	2.78	2.82	2.35	0.45 (0.252)
Somatization	−2.27*	1.75	2.13	1.06	1.25	0.40 (0.228)
Social stress	−2.13*	3.26	3.16	1.91	2.79	0.45 (0.348)
Anxiety	−1.24	8.29	3.36	7.44	3.27	0.26 (0.142)
Depression	−2.43*	3.06	3.52	1.53	2.86	0.48 (0.266)
Sense of inadequacy	−2.60*	4.82	3.09	3.20	2.88	0.54 (0.296)
**Personal adjustment**						
Interpersonal relations	0.98	14.00	2.56	14.50	2.07	−0.21 (0.120)
Relations with parents	3.39***	7.31	2.09	8.44	0.96	−0.69 (0.376)
Self-esteem	2.63**	5.85	2.59	6.97	1.80	−0.50 (0.274)
Self-confidence	2.09*	6.36	1.41	6.94	1.15	−0.45 (0.252)
**School adjustment**						
Negative attitude school	−1.39	3.44	2.87	2.62	2.56	0.30 (0.166)
Negative attitude teachers	−1.45	4.09	2.68	3.27	2.76	0.32 (0.182)
Sensation seeking	−2.23*	5.28	3.39	3.79	2.84	0.48 (0.266)
**Global indexes**						
Clinical maladjustment	−2.67**	212.5	38.88	195.2	28.21	0.51 (0.282)
Personal adjustment	2.87**	181.1	40.9	206.8	27.71	−0.74 (0.398)
School maladjustment	−2.02*	158.8	27.4	147.2	26.68	0.43 (0.236)
Emotional symptoms	−2.46*	322.4	58.9	293.0	48.83	0.54 (0.304)

df(107); M_*APV*_, mean victimized child-parental violence adolescent group; SD_*APV*_, standard deviation victimized child-parental violence adolescent group; M_*n*–*APV*_, mean victimized non-aggressor adolescent group; SD_*n*–*APV*_, standard deviation victimized non-aggressor adolescent group; d, Cohen’s d; PS_*ES*_, Probability of Superiority of the Effect Size. ***p < 0.001, **p < 0.01, *p < 0.05.

## Discussion

In this study on a community sample, APV was highly prevalent both toward the mother (51.4%) and father (51.9%), with negligible physical violence (1.9%), which agreed with the findings of previous studies (Gámez-Guadix and Calvete, 2012; Ibabe et al., 2013; Aroca-Montolío et al., 2014; Calvete et al., 2015). Contrary to previous studies reporting higher rates of violence against mothers than fathers (Condry and Miles, 2014; Holt, 2016; Simmons et al., 2018), the results did not show differences between mothers and fathers for either the gender of the child or for the type of violence. Regarding this finding, some authors have proposed a link between the victim’s gender and gender roles (Cottrell and Monk, 2004; Gallagher, 2004; Cortina and Martín, 2020). Thus, it is possible that the incorporation of women to the labor market, along with a dynamic flexible family model (Buehler, 2020), may contribute to a parity between mothers and fathers as victims (Williams et al., 2017). Furthermore, the blurring of gender roles in adolescents in comparison to traditional gender roles of the past may also play a key role.

As for the characteristics of adolescents involved in APV, no significant gender differences were observed. In general, there is no consensus in the literature regarding differences between boys and girls in exercising APV (Moulds and Day, 2017), with some studies reporting males perpetrate more violence than females (Ibabe et al., 2014; Calvete et al., 2015; Kuay et al., 2016), whereas others studies found no genders differences (Calvete et al., 2014; Margolin and Baucom, 2014; Bartle-Haring et al., 2015). The lack of consensus is associated to the type of samples employed, judicial or normalized. In this sense, the result of the present study corroborated the findings of previous studies in community samples where gender differences were blurred (Loinaz et al., 2020). Likewise, in the analysis of other types of violence related to adolescence, a number of studies have found no differences between boys and girls (Marcos et al., 2020), and have questioned the relevance of gender socialization in this particular phenomena.

Moreover, the results corroborated greater maladjustment in adolescents who engaged in psychological and/or physical violence toward their parents (Ibabe et al., 2013; Ibabe, 2014) in significant areas of functioning (e.g., psychological, personal, and school) of their lives (Seijo et al., 2016). Whether maladjustment causes violent behavior or inversely, the latter causes maladjustment, is an issue that goes beyond the scope of this study as the methodology was not designed to establish the causality of this relationship. Nevertheless, the findings underscored the need for multimodal and multilevel prevention, in accordance with the non-model approach (Arce et al., 2014; Basanta et al., 2018). Thus, it is of vital importance to determine the precise personal, family, and social needs for the psychological adjustment of adolescents involved in APV, and to estimate the magnitude in order to design and develop efficacious prevention programs and interventions (Mayorga et al., 2020).

The scientific literature has highlighted the importance of parenting in terms of parental socialization styles in generating and maintaining APV (Laurent and Derry, 1999; Cottrell and Monk, 2004; Contreras and Cano, 2014; Calvete et al., 2015; Suárez-Relinque et al., 2019). The loss of parental authority, lack of discipline and consistent norms, and poor affection and support were characteristics of families exposed to APV (Ibabe et al., 2013; Calvete et al., 2015). The results showed that adolescents engaged in APV reported higher levels of strictness and supervision both in paternal and maternal parenting. Furthermore, they reported more indifference and less affect, acceptance, and involvement in childrearing only in the maternal parenting style (Aroca-Montolío et al., 2012; Contreras and Cano, 2014; Calvete et al., 2015; Ibabe, 2015). The results corroborated the literature regarding the importance of mother positive parenting as a protective factor (Kawabata et al., 2011), however, they do not support this relationship for a father parenting style, giving more relevance to the mother’s one. Furthermore, in accordance with the current approach of Positive Parenting of the Council of Europe (Council of Europe, 2006), the conception of traditional parenting associated to authority, discipline, and obedience should be replaced by the broader concept of parental responsibility (Fariña et al., 2017), which is particularly aimed at satisfying the needs of adolescents and safeguarding their rights and wellbeing, ensuring respect for parents, and analyzing specific parenting techniques and the quality of child parent relations (Simmons et al., 2018). In this way, the affectivity and quality of family relationships are essential to prevent the development and maintenance of APV (Contreras and Cano, 2014; Ibabe and Bentler, 2016; Beckmann et al., 2017; Suárez-Relinque et al., 2019). Therefore, programs aimed at parental warmth are recommended (Bisby et al., 2017; Curtis et al., 2019).

The exposure to family violence as a variable linked to APV has been well documented in the literature (Ibabe et al., 2013; Loinaz et al., 2018). Recent studies have revealed that both direct and vicarious victimization were directly related to growing violence (Kennedy et al., 2010; Ibabe, 2015; Izaguirre and Calvete, 2017; Gallego et al., 2019). The results of the present study have corroborated this relationship with a large effect size. However, the results should be interpreted with caution given that the transversal design of this study was not designed to establish causal relations, and in spite of delimiting the temporal criterion of the previous year in applying the measures, it was impossible to determine the dynamics of violent relations if the violent behavior of adolescents was a reactive response to victimization or if the violent behavior of parents was a response to the violent behavior of adolescents (Brezina, 1999). Thus, APV should be assessed through the simultaneous analysis of growing violence and parent-to-child violence (Seijo et al., 2016), and growing violence as a predictor of parent-to-child violence (Gallego et al., 2019).

The presence of violent dynamics in the family should be considered a risk factor for the development of adolescents (Loinaz et al., 2018; Schmidt et al., 2018) and stifles and/or negatively influences the adjustment of adolescents in a range of significant areas of functioning. The results showed that adolescents who have suffered victimization and engaged in APV exhibited a higher psychological, personal, and school maladjustment (Castañeda et al., 2012; Ibabe, 2014; Rosado et al., 2017), in comparison to adolescents who did not exercise growing violence. According to several publications of the American Academy of Pediatrics, victimization in the family is considered to be an adverse childhood experience, a risk to health, and for the positive development of the adolescents (Garner et al., 2012; Exner-Cortens et al., 2013). As a toxic stress factor, it activates extreme and long lasting physiological responses to stress (Ecological and Biological Development Perspective). This exposure causes psychological injury and has negative implications on the physical and psychological development of adolescents (Garner et al., 2012; Exner-Cortens et al., 2013; Corrás et al., 2017).

Nonetheless, the results of the present study are subjected to limitations concerning generalizations: the sample size, transversal study design, and self-report of victimization may be biased by defensiveness—underreporting response bias (Harbin and Madden, 1979; Arce et al., 2015a). Further research to examine the different systems involved in APV (Cottrell and Monk, 2004), and to establish a measure of APV with clearly defined strict criteria is needed (Gallego et al., 2019). It is worth noting that none of the APV measurement instruments available evaluates recidivism that is a critical aspect, particularly in terms of psychological violence, the intent to cause harm, nor the injury caused (Arce et al., 2015b).

## Data Availability Statement

The raw data supporting the conclusions of this article will be made available by the authors, without undue reservation.

## Ethics Statement

Ethical review and approval was not required for the study on human participants in accordance with the local legislation and institutional requirements. Written informed consent to participate in this study was provided by the participants’ legal guardian/next of kin.

## Author Contributions

All authors have participated in the collection of the participants’ data and managed their statistical treatment, and proceeded to the writing of the manuscript. DS, MN, and RG have directed all the work, participated in the interpretation and discussion of the data, as well as made interesting intellectual contributions in relation to the elaboration of the conclusions. Finally, all the signatories, one by one, have approved the final version of the manuscript for publication. Therefore, all authors are responsible and guarantee that all aspects that make up the manuscript have been reviewed and discussed among the authors in order to be exposed with the maximum precision and integrity.

## Conflict of Interest

The authors declare that the research was conducted in the absence of any commercial or financial relationships that could be construed as a potential conflict of interest.
